# Proteomics shows that brain metastases of lung adenocarcinoma overexpress ribosomal proteins in response to gamma knife radiosurgery

**DOI:** 10.1038/s41598-024-58967-y

**Published:** 2024-07-08

**Authors:** Luqing Tong, Ke Ye, Qun Chen, Xiaoxi Wang, Chi Hu, Qingsheng Xu, Lihui Zhou, Renya Zhan, Ying Tong

**Affiliations:** 1 Department of Neurosurgery, The First Affiliated Hospital, Zhejiang University School of Medicine, No.79 Qingchun Street, Shangcheng District, Hangzhou, 310003 Zhejiang China; 2 Department of Pathology, The First Affiliated Hospital, Zhejiang University School of Medicine, No.79 Qingchun Street, Shangcheng District, Hangzhou, 310003 Zhejiang China; 3 Gamma Knife Centre, The First Affiliated Hospital, Zhejiang University School of Medicine, No.79 Qingchun Street, Shangcheng District, Hangzhou, 310003 Zhejiang China

**Keywords:** Brain metastasis, Lung adenocarcinoma, Gamma knife, Proteomics, Ribosome, Radiation resistance, Metastasis, Radiotherapy, Prognostic markers, Proteomics

## Abstract

Gamma knife radiosurgery (GKRS) is recommended as the first-line treatment for brain metastases of lung adenocarcinoma (LUAD) in many guidelines, but its specific mechanism is unclear. We aimed to study the changes in the proteome of brain metastases of LUAD in response to the hyperacute phase of GKRS and further explore the mechanism of differentially expressed proteins (DEPs). Cancer tissues were collected from a clinical trial for neoadjuvant stereotactic radiosurgery before surgical resection of large brain metastases (ChiCTR2000038995). Five brain metastasis tissues of LUAD were collected within 24 h after GKRS. Five brain metastasis tissues without radiotherapy were collected as control samples. Proteomics analysis showed that 163 proteins were upregulated and 25 proteins were downregulated. GO and KEGG enrichment analyses showed that the DEPs were closely related to ribosomes. Fifty-three of 70 ribosomal proteins were significantly overexpressed, while none of them were underexpressed. The risk score constructed from 7 upregulated ribosomal proteins (RPL4, RPS19, RPS16, RPLP0, RPS2, RPS26 and RPS25) was an independent risk factor for the survival time of LUAD patients. Overexpression of ribosomal proteins may represent a desperate response to lethal radiotherapy. We propose that targeted inhibition of these ribosomal proteins may enhance the efficacy of GKRS.

## Introduction

Brain metastases (BMs) are the most common type of intracranial neoplasm in adults. BMs occur in up to 40% of patients with lung adenocarcinoma (LUAD)^1^, which is the most common cause of BMs. Many guidelines recommend stereotactic radiosurgery (SRS) for BMs, among which gamma knife radiosurgery (GKRS) is the most widely used^2,3^.

Few studies have investigated the mechanism associated with the hyperacute phase of GKRS. This is probably due to the difficulty in obtaining patient tissue during this phase. Most patients are not operated on again within several months after GKRS treatment. Mechanistically, GKRS treatment involves a pattern of radiotherapy that focuses gamma rays on the target tissue and causes radiation damage. The main mechanism of radiotherapy produced by different rays (including gamma rays) is similar: ionizing radiation (IR) can directly cause DNA damage or indirectly cause DNA damage by generating free radicals from water and other substances in cells. The oxygen fixation hypothesis holds that oxygen reacts with free radicals to form organic peroxy radicals, which immobilize the target substance in the form of irreversible ROOH. Therefore, hypoxic tissues are more radiation tolerant^4^. Although IR may cause various types of DNA damage, double-strand breaks are considered to be the most critical type. Double-strand breaks are very difficult for cells to repair, and the repair process can produce abnormal chromosomes that lead to mitotic disaster or mutations that reduce replication adaptability^5^. Cancer cells, which are highly proliferative and often have impaired DNA repair ability^6^, should be more sensitive than noncancer cells to lethal radiation. Cell death in the form of reproductive death^7,8^, interphase death^7^, apoptosis^9^, autophagy^10^, necrosis^11^, etc., does not occur at the moment of IR administration but approximately hours to tens of days after radiation exposure. Cells are likely to undergo a series of biochemical reactions between the time when cells are exposed to radiation and the time when most cells finally die. Compared to the primary non-irradiated tumor, tumor necrosis increased and multiple immunomodulatory cell populations (CD3+, CD4+, and CD8+) reduced when the brain metastases underwent preoperative SRS within median interval of 67.8 h (18.25–160.61 h)^12^. Positron Emission Tomographic scans using [18F]-fluorodeoxyglucose (PET-FDG) showed the glucose uptake ratio increased 25% to 42% 1 day post-SRS, then decreased to between 10% above and 12% below the baseline value 7 days post-SRS^13^. More research on the mechanism of hyperacute effect of GKRS is needed.

In this study, proteomics analysis was performed to explore the biochemical processes associated with the hyperacute phase of GKRS. LUAD BM tissues were collected on the day after GKRS treatment. Cancer tissues were collected from patients in a clinical trial of neoadjuvant stereotactic radiosurgery before surgical resection of large BMs (ChiCTR2000038995).

## Materials and methods

### Patients, tissues and datasets

A clinical trial (ChiCTR2000038995) of neoadjuvant stereotactic radiosurgery before surgical resection of large BMs was registered to evaluate the safety and effectiveness of preoperative GKRS therapy.

The BM tissues of LUAD patients were immediately fixed with 4% paraformaldehyde (P1110, Solarbio, China) after surgery. Then, they were embedded in paraffin within one month after surgery. The BMs that received GKRS therapy were classified as the post-GKRS group, and those that did not receive GKRS therapy were classified as the no-GKRS group. In total, 6 post-GKRS tissues and 10 no-GKRS tissues were collected. According to the tissue size, burn degree and the number of blood cells, the 5 post-GKRS tissues and 5 no-GKRS tissues were selected for proteomics detection. Patient sex, patient age, the latest MR image before surgery, pathological type, molecular subtypes, GKRS dosage, and other preoperative treatments were recorded.

The RNA sequencing dataset of LUAD from TCGA (https://portal.gdc.cancer.gov/) was used to evaluate the possible prognostic value of the upregulated ribosomal proteins. Transcripts per kilobase million (TPM) data were selected. The latest clinical prognosis data had been updated by LIU Jianfang et al.^31^.

### Hematoxylin–eosin (HE) staining

HE staining was conducted according to the manufacturer’s instructions. Sections were stained with HE (G1120, Solarbio, China) followed by dehydration in ethanol (A112717, Aladdin, China) and xylene (X139941, Aladdin, China). Images were obtained with a full automatic digital slice scanning system (KF-PRO-120, KFBIO, China).

### LC‒MS/MS

The 10 paraffinized tissues were submitted to Oebiotech (Shanghai, China) for LS/MS detection. The basic process of the label-free proteomics experiment was as follows: total protein in the sample was extracted, part of the sample was used to determine protein concentrations and to carry out SDS‒PAGE detection, and the other part of the sample was subjected to trypsin enzymatic hydrolysis. After desalting the enzymolysis peptide segment, LC‒MS/MS analysis and data analysis were conducted.

Nanoflow reversed-phase chromatography was performed with an EASY-nLC 1200 system (Thermo Fisher Scientific, USA). Liquid chromatography was coupled online to a hybrid TIMS quadrupole TOF mass spectrometer (Bruker timsTOF Pro, Germany) via a Captive Spray nano-electrospray ion source.

### Bioinformatics and statistics

The online OECloud tool (https://cloud.oebiotech.com/task) was used to perform bioinformatic analysis and export the proteomics results. The expression data of all credible proteins were submitted to the Oebiotech platform. Principal coordinate analysis (PCA) was performed by using the Bray–Curtis dissimilarity metric. Differentially expressed proteins (DEPs) were screened as follows: fold change ≥ 1.2 or fold change ≤ 1/1.2 and a t test p value < 0.05. The DEPs are shown in a volcano plot and heatmap. The color of the heatmap indicates the value of log2 (expression + 1). The top 15 GO enrichment and KEGG enrichment terms related to these DEPs are shown in bubble plots.

Another online tool, Xiantao Scholarship (https://www.xiantao.love), was used to perform bioinformatic analysis and export the figures for the TCGA dataset. Tenfold cross-validation and the R package glmnet (4.1-2 version) and survival (3.2-10 version) were used to screen a lasso coefficient. The lambda.min value was used as the cutoff. A Lasso variable trajectory diagram was also produced with the R package glmnet (4.1-2 version) and survival package (3.2-10 version). The ggplot2 package (version 3.3.3) was used to draw forest plots, grouped expression plots, and risk score diagrams. The survminer package (0.4.9 version) and survival package (3.2-10 version) were used to produce survival curves. The Rms package (6.2-0 version) and survival package (3.2-10 version) were used to draw the nomogram and the nomogram calibration plot. The survival (version 3.2-10) package was used to perform univariate Cox regression analysis and multivariate Cox regression analysis. The factors with a p value < 0.10 (7 factors) in univariate Cox regression analysis were uploaded for multivariate Cox regression analysis. Multivariate Cox regression analysis resulted in 7 EXP(β)s and an intercept (− 3.043762655). Then, every case get a risk score =  + 0.149378894*Exp(RPL4) + 0.077032501*Exp(RPS19) + 0.009953769*Exp(RPS16) + 0.032681042*Exp(RPLP0) + 0.091708032*Exp(RPS2) − 0.083562796 *Exp(RPS25) + 0.062949989*Exp(RPS26) − 3.043762655. The ComplexHeatmap (2.13.1 version) package was used to draw the heatmap of detected ribosomal proteins.

### Ethics approval and consent to participate

This study was carried out in accordance with the principles of the Helsinki Declaration and approved by the Clinical Research Ethical Committee of the First Affiliated Hospital, Zhejiang University School of Medicine (IIT20220842A). Written informed consent was obtained from all donors or their relatives.

## Results

A clinical trial (ChiCTR2000038995) of neoadjuvant stereotactic radiosurgery before surgical resection of large BMs was registered to evaluate the safety and effectiveness of preoperative GKRS therapy. The tissues of BMs of LUAD patients were obtained during this trial.

BMs that received GKRS therapy were classified as the post-GKRS group, and those that did not receive GKRS therapy were classified as the no-GKRS group. According to the tissue size, burn degree and the number of blood cells, 5 post-GKRS and 5 no-GKRS tissues were selected for proteomics detection. There was no significant difference between the two groups of patients in terms of sex, age, mutation, targeted therapy, or chemotherapy (Table 1). Before the enrollment in this trial, no patient had received radiotherapy. The 5 patients of post-GKRS group underwent GKRS within median interval of 18 h (16–22 h). Four patients underwent a median preoperative GKRS dose of 15.75 Gy (15–18 Gy) in 1 fraction, with the other patient (G1006) receiving 24 Gy in 3 fractions.

**Table 1:** Characteristics of the selected cases.

Characteristics	Post-GKRS	No-GKRS	p value
n	5	5	
Gender, n (%)			1.000
Male	3 (30%)	3 (30%)	
Female	2 (20%)	2 (20%)	
Age, mean ± sd	58.913 ± 12.492	58.661 ± 11.377	0.974
Mutation, n (%)			1.000
EGFR	3 (30%)	2 (20%)	
No detection	2 (20%)	1 (10%)	
ROS1	0 (0%)	1 (10%)	
ALK	0 (0%)	1 (10%)	
Targeted therapy, n (%)			1.000
Yes	2 (20%)	3 (30%)	
No	3 (30%)	2 (20%)	
Chemotherapy, n (%)			1.000
No	4 (40%)	4 (40%)	
Yes	1 (10%)	1 (10%)	

### MR and HE staining

**Figure 1:**
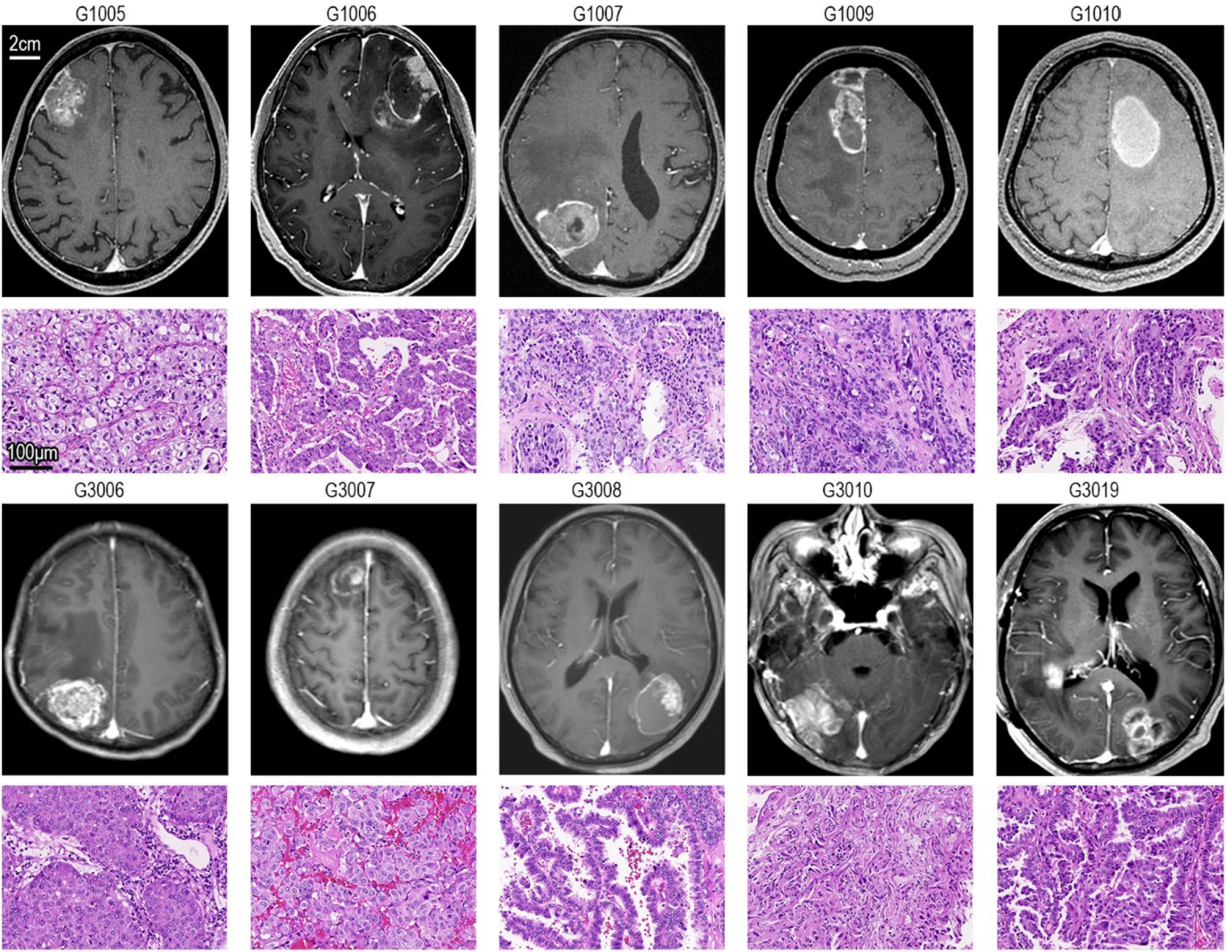
MR images and HE staining images. The post-γ group consisted of G1005, G1006, G1007, G1009, and G1010. The no-γ group consisted of G3006, G3007, G3008, G3010, and G3019. The MR images with the largest neoplastic diameter were selected. HE staining images are also shown under the MR images for every case.

The MR images and HE staining images of the tissue samples are shown in Fig. 1. The post-GKRS group samples were labelled G1005, G1006, G1007, G1009, and G1010. The no-GKRS group samples were labelled G3006, G3007, G3008, G3010, and G3019. Mixed signals, which indicated malignant neoplasms, were shown by gadolinium-enhanced MR for all patients. Low signal intensity around the BMs, which indicated edema, appeared in all cases to varying degrees. HE staining indicated that these tissues were metastatic adenocarcinomas.

### Proteins upregulated by GKRS therapy were enriched in ribosomal proteins

**Figure 2:**
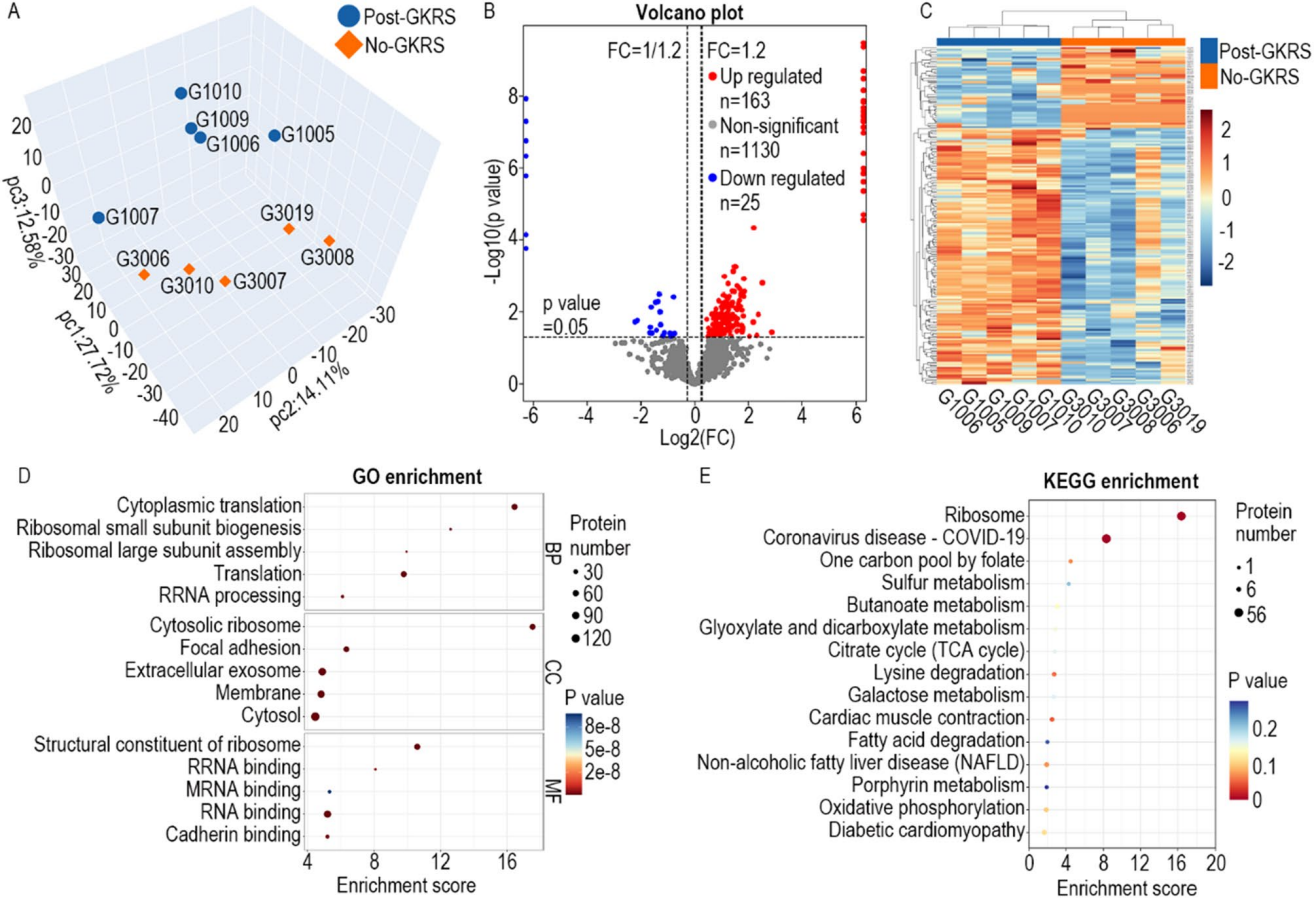
Proteins upregulated by gamma-knife therapy were enriched in ribosomal proteins. (**A**) PCA score chart. Each point in the chart represents a sample. The distance between different samples can be seen visually, and the similarity or difference in the samples can be judged accordingly. (**B**) The volcano plot shows 25 downregulated proteins (blue) and 163 upregulated proteins (red). (**C**) Heatmap showing that the DEPs clustered into the correct groups. (**D**) Top 5 biological process (BP), cellular component (CC), and molecular function (MF) terms. (**E**) Top 15 KEGG pathway enrichment terms. FC, fold change.

Proteomics analysis revealed 1833 credible proteins. Principal component analysis (PCA) was carried out based on the expression of credible proteins to show the relationship among samples in different dimensions (Fig. 2A). If the difference between two samples is significant, the two coordinate points are relatively far away on the score chart. Although the samples in each group differed from each other, the 10 samples were roughly classified into two groups according to their GKRS history. Difference analysis revealed that 25 proteins were downregulated and 163 proteins were upregulated by GKRS treatment (Fig. 2B). Cluster analysis showed that the clusters of these differentially expressed proteins (DEPs) were consistent with the PCA results (Fig. 2C).

GO enrichment and KEGG pathway enrichment were carried out to explore the possible functions of these DEPs. Based on biological process (BP) analysis, these DEPs were enriched in cytoplasmic translation, ribosomal small subunit biogenesis, ribosomal large subunit assembly, translation, and rRNA processing. For cellular component (CC) analysis, they were enriched in cytosolic ribosome, focal adhesion, extracellular exosome, membrane, and cytosol. for According to molecular function (MF) analysis, they were enriched in structural constituent of ribosome, rRNA binding, mRNA binding, RNA binding, and cadherin binding. All top 5 BP terms and top 4 MF terms were related to the ribosome-centered translation process (Fig. 2D). KEGG pathway enrichment showed that these DEPs were mainly enriched in ribosomal proteins (Fig. 2E).

**Figure 3:**
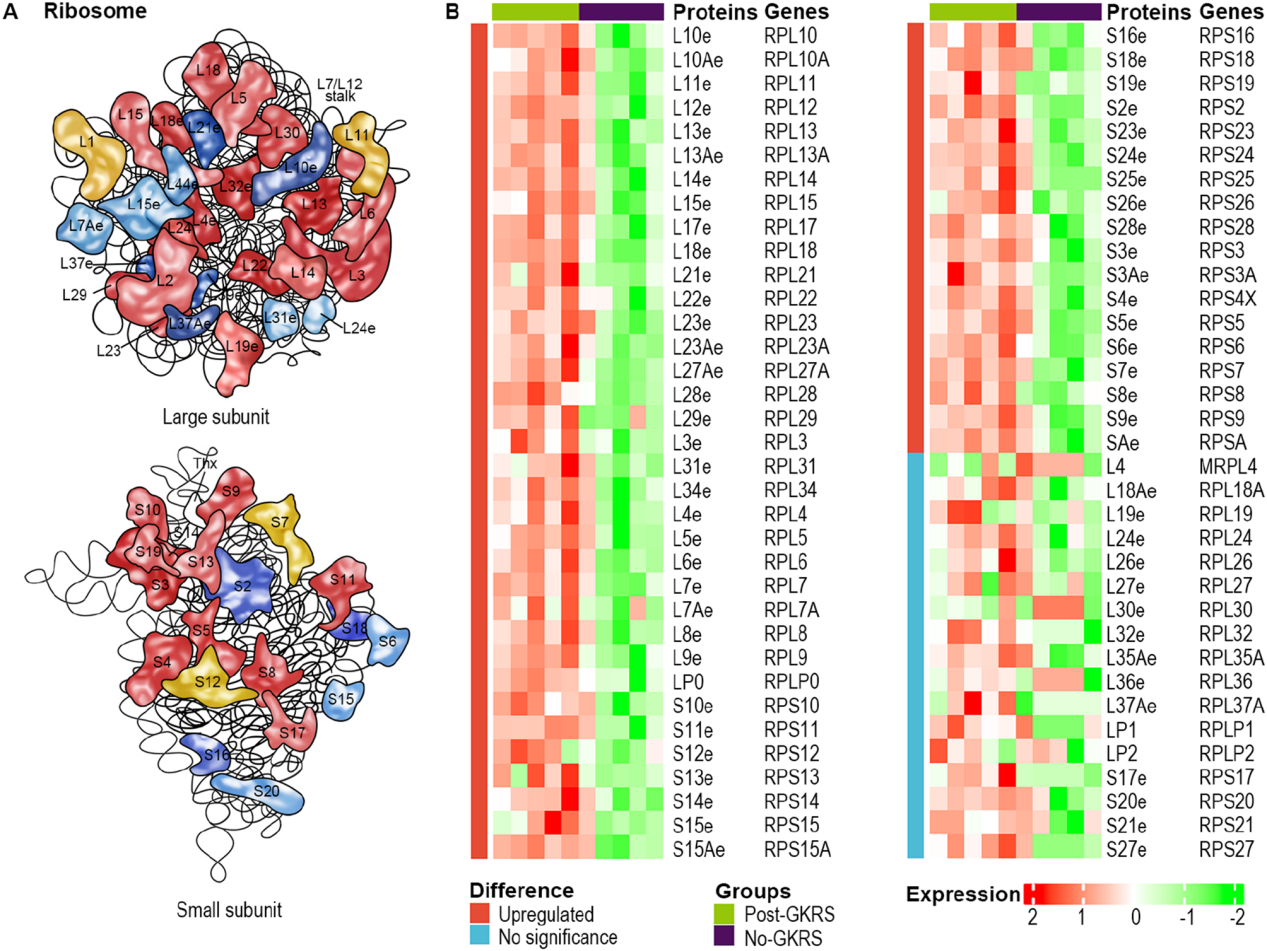
The majority ribosomal proteins were upregulated by GKRS therapy. (**A**) The map of the ribosomal pathway of Homo sapiens (https://www.genome.jp/entry/hsa03010). (**B**) Heatmaps of the 70 detected ribosomal proteins. The orange column showed the 53 ribosomal proteins were significantly upregulated by γ-knife. The light blue column showed no difference between the 2 groups. Relative expression levels were stained by red and green.

According to the KEGG pathway hsa03010, 134 proteins were involved in the ribosomal pathway (Fig. 3A). In this study, proteomics detected 70 ribosomal proteins out of the 134 ribosomal proteins. In addition, 53 of the 70 (75.7%) ribosomal proteins were significantly upregulated by GKRS therapy (Fig. 3B). The expression levels of the remaining 17 (24.3%) ribosomal proteins were not significantly altered by GKRS treatment. No ribosomal protein was significantly downregulated. Actually, most of the remaining 17 (24.3%) ribosomal proteins were upregulated by GKRS therapy but without significance (Fig. 3B). More ribosomal proteins could be confirmed to increase expression levels if more samples are detected. This indicates that the expression level of ribosomal proteins is overwhelmingly upregulated after GKRS treatment.

### Proteins upregulated by GKRS therapy were associated with a poor prognosis

A previous study indicated that gamma radiation-upregulated ribosomal proteins could be used to augment radiation resistance by increasing the cell’s ability to produce proteins involved in recovery^14^. Thus, the expression levels of ribosomal proteins may be associated with the prognosis of patients with lung cancer. Here, we explored the relationship between the expression levels of 53 upregulated ribosomal proteins and the overall survival of lung cancer patients by analyzing the RNA sequencing dataset of LUAD from The Cancer Genome Atlas (TCGA).

**Figure 4:**
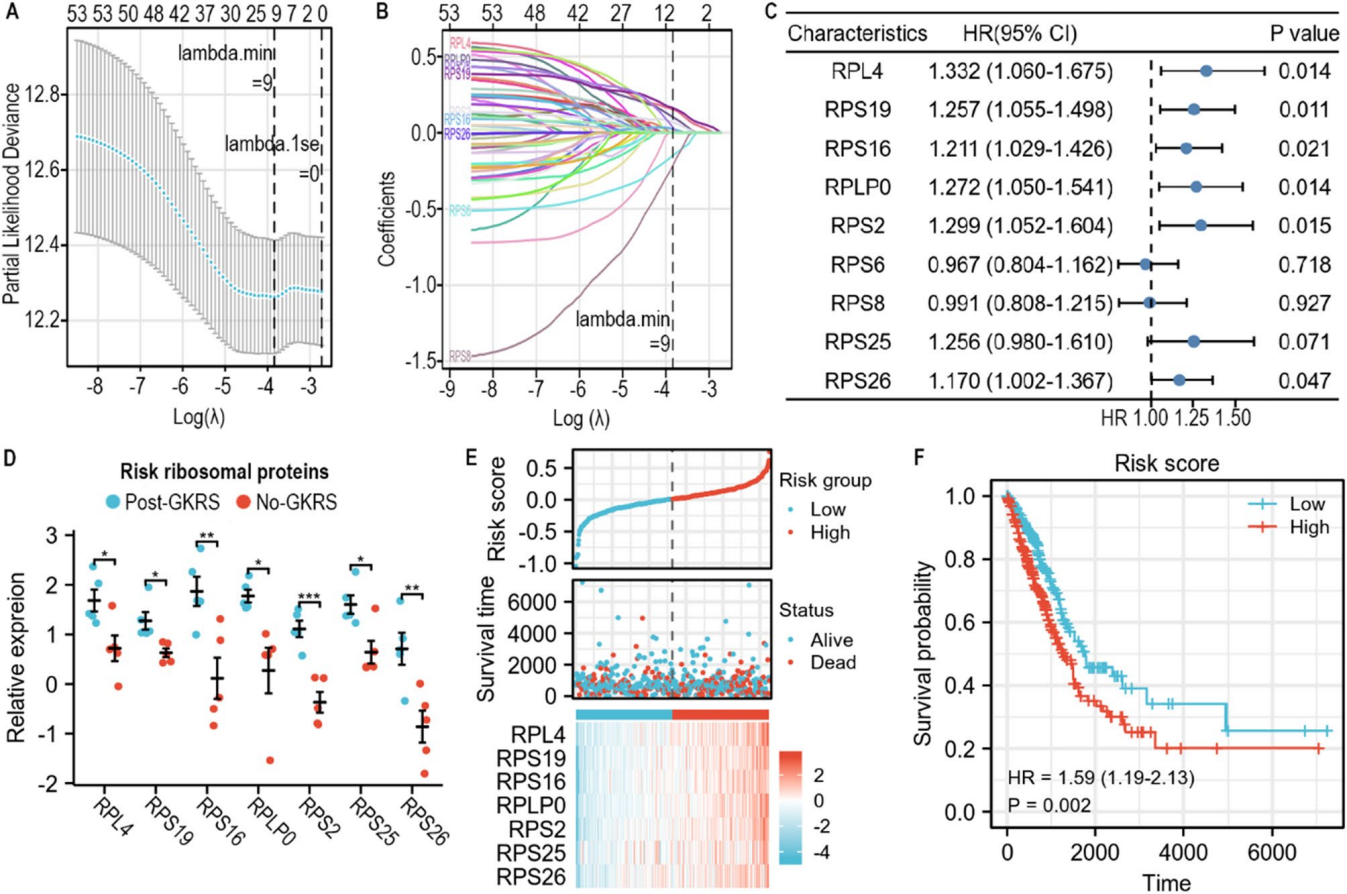
Identification of key ribosomal proteins. (**A**) The lambda.min value of lasso coefficient was used as cut-off. (**B**) The potential prognostic proteins were determined by lasso variable trajectory diagram. (**C**) Key ribosomal proteins were identified by univariate cox regression analysis with p value < 0.1. RPL4, RPS19, RPS16, RPLP0, RPS2, RPS26 and RPS25 had close relation to overall survival of LUAD patients. (**D**) The relative expression of the 7 key ribosomal proteins. (**E**) The relationship between risk score and survival time, expression levels of 7 key ribosomal proteins. (**F**) The survival curve of risk score.

The Lasso coefficient was screened by tenfold cross-validation to obtain potential prognostic proteins. The lambda.min value was 9 and was used as the cutoff (Fig. 4A). The Lasso variable trajectory diagram showed that the potential prognostic proteins were ribosomal protein (RP) L4 (RPL4), RPS19, RPS16, RPLP0, RPS2, RPS6, RPS8, RPS25, and RPS26 (Fig. 4B). Univariate Cox regression analysis suggested that the overall survival of LUAD patients was closely related to the expression levels of RPL4, RPS19, RPS16, RPLP0, RPS2, and RPS26 (Fig. 4C and Table 2). RPS25, with a p value of 0.071, was also included in the multivariate Cox regression analysis. However, multivariate Cox regression analysis suggested that none of these proteins were independent risk factors for the prognosis of LUAD patients (Table 2).

**Table 2:** Cox regression analysis of potential prognostic proteins.

Characteristics	Total (N)	Univariate analysis		Multivariate analysis	
		Hazard ratio (95% CI)	p value	Hazard ratio (95% CI)	p value
RPL4	526	1.332 (1.060–1.675)	**0.014**	1.161 (0.807–1.670)	0.420
RPS19	526	1.257 (1.055–1.498)	**0.011**	1.080 (0.748–1.559)	0.681
RPS16	526	1.211 (1.029–1.426)	**0.021**	1.010 (0.757–1.347)	0.946
RPLP0	526	1.272 (1.050–1.541)	**0.014**	1.033 (0.740–1.444)	0.848
RPS2	526	1.299 (1.052–1.604)	**0.015**	1.096 (0.788–1.525)	0.587
RPS6	526	0.967 (0.804–1.162)	0.718		
RPS8	526	0.991 (0.808–1.215)	0.927		
RPS25	526	1.256 (0.980–1.610)	0.071	0.920 (0.638–1.327)	0.655
RPS26	526	1.170 (1.002–1.367)	**0.047**	1.065 (0.879–1.290)	0.519

Significant values are in [bold].

Multivariate Cox regression analysis resulted in the following prognostic formula: risk score =  + 0.149378894*Exp(RPL4) + 0.077032501*Exp(RPS19) + 0.009953769*Exp(RPS16) + 0.032681042*Exp(RPLP0) + 0.091708032*Exp(RPS2) − 0.083562796 *Exp(RPS25) + 0.062949989*Exp(RPS26) − 3.043762655. Each case received a risk score. The relative expression levels of key proteins in BMs are shown in Fig. 4D. All of these proteins were significantly upregulated by gamma radiation. The prognostic value of the risk score was compared according to age, sex, primary outcome of standard therapy, distant metastasis, lymph node metastasis, tumor size, stage, pack-years smoked, and residual tumor status. Multivariate Cox regression analysis suggested that the primary outcome of standard therapy and the risk score were independent risk factors for the overall survival time of LUAD patients (Table 3). With an increase in the risk score, the mortality rate of patients increased and the survival rate decreased (Fig. 4E). Log rank survival analysis also showed that the risk score distinguished the overall survival time of LUAD patients (Fig. 4F).

**Table 3:** Cox regression analysis of potential prognostic factors.

Characteristics	Total (N)	Univariate analysis		Multivariate analysis	
		Hazard ratio (95% CI)	p value	Hazard ratio (95% CI)	p value
Age	481	0.986 (0.969–1.004)	0.120		
Gender	491				
MALE	229	Reference			
FEMALE	262	1.011 (0.702–1.455)	0.954		
Primary_outcome	404				
CR or PR	299	Reference			
Stable disease	36	1.823 (0.856–3.884)	0.120	1.438 (0.653–3.163)	0.367
Progressive disease	69	5.974 (3.893–9.169)	** < 0.001**	5.268 (3.188–8.705)	** < 0.001**
M	491				
M0	323	Reference			
M1	21	2.424 (1.253–4.686)	**0.009**	0.665 (0.184–2.405)	0.534
MX	147	0.942 (0.611–1.452)	0.786	1.076 (0.626–1.850)	0.791
N	491				
N2 or N3	65	Reference			
N0	327	0.380 (0.233–0.617)	** < 0.001**	1.063 (0.284–3.972)	0.928
NX	16	0.324 (0.076–1.376)	0.127	0.000 (0.000–Inf)	0.994
N1	83	1.044 (0.618–1.763)	0.872	1.306 (0.440–3.873)	0.631
T	491				
T1	168	Reference			
T2	262	1.700 (1.084–2.666)	**0.021**	0.943 (0.559–1.590)	0.825
T3	43	2.849 (1.455–5.579)	**0.002**	0.858 (0.313–2.355)	0.766
T4	15	2.772 (1.062–7.234)	**0.037**	0.784 (0.190–3.229)	0.736
TX	3	7.650 (1.796–32.589)	**0.006**	4,691,055.754 (0.000–Inf)	0.994
Stage	491				
Stage I	277	Reference			
Stage II	112	3.031 (1.939–4.738)	** < 0.001**	2.149 (0.891–5.186)	0.089
Stage III or IV	94	3.625 (2.315–5.678)	** < 0.001**	3.412 (0.816–14.258)	0.093
Stage X	8	1.580 (0.381–6.549)	0.529	0.976 (0.199–4.777)	0.976
Pack_years_smoked	336	1.003 (0.994–1.013)	0.448		
Residual_tumor	491				
R0	318	Reference			
R1 or R2	13	4.795 (2.384–9.647)	** < 0.001**	2.114 (0.819–5.459)	0.122
RX	160	0.936 (0.611–1.435)	0.761	0.649 (0.377–1.117)	0.119
RiskScore	491	3.303 (1.374–7.936)	**0.008**	3.505 (1.129–10.885)	**0.030**

Significant values are in [bold].

**Figure 5:**
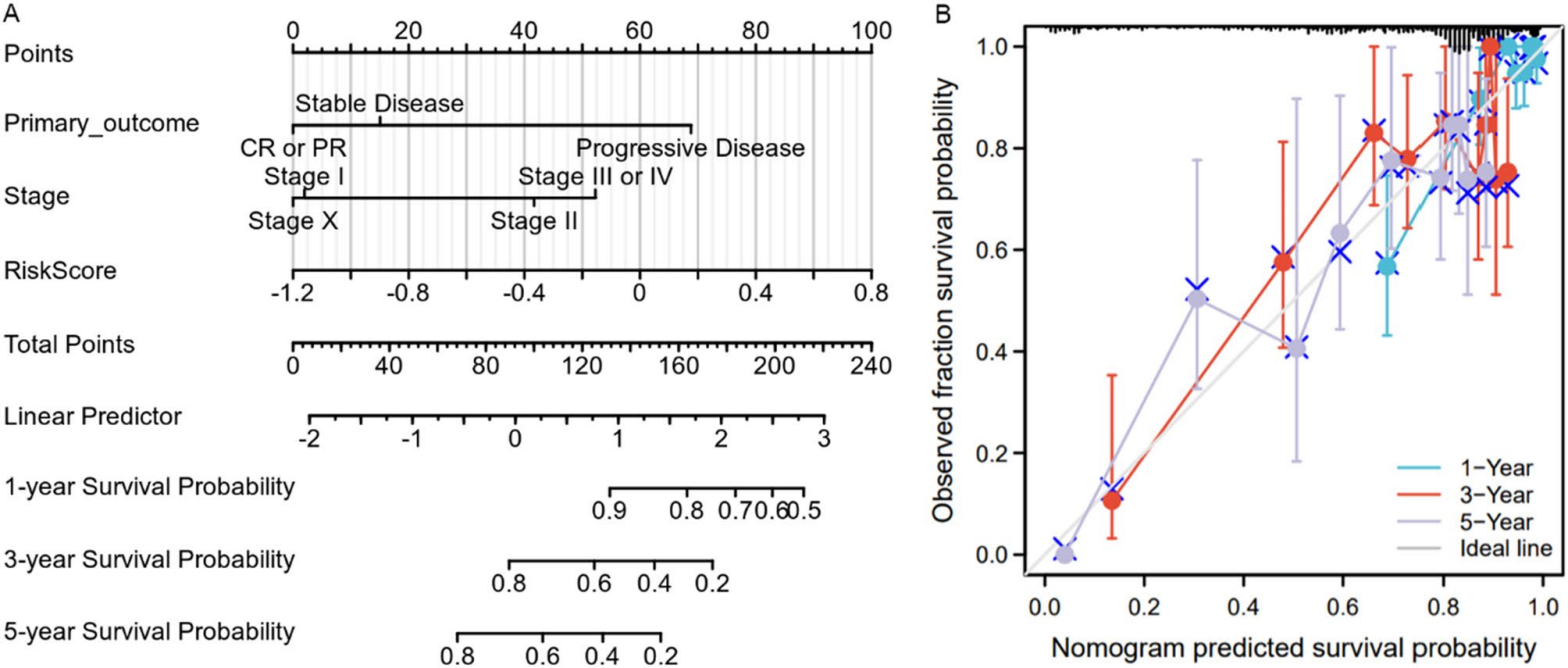
A nomogram for LUAD patients. (**A**) A nomogram constructed by risk score, primary come-out of standard therapy and stage. This nomogram can predict the 1 year’s, 3 years’, and 5 years’ survival of LUAD patients. (**B**) A nomogram calibration plot for the nomogram.

Multivariate Cox regression analysis also suggested that stage was nearly an independent risk factor for the overall survival time of LUAD patients (Table 3). Here, the risk score, primary outcome of standard therapy and stage were used to construct a nomogram to predict the 1-year, 3-year, and 5-year survival outcome (Fig. 5A). Calibration analysis was carried out to assess the predictive ability of the nomogram. The nomogram calibration plot (Fig. 5B) indicated that the nomogram was well calibrated, with mean predicted probabilities for each subgroup close to observed probabilities.

## Discussion

Ribosomal protein levels were significantly increased in the acute stage of IR therapy. Single-cell transcriptomics analysis revealed that DEGs upregulated by IR in breast cancer cells were highly enriched in GO terms associated with ribosomes (including the GO terms ribosome assembly and ribosome biogenesis)^15^. Fibroblasts harvested from cultures 2 h after exposure to 2 Gy of gamma radiation showed instantaneous enrichment in a gene set belonging to ribosomes^16^. A large increase in ribosomal content was invoked by acute gamma radiation exposure in the black yeast *Exophiala dermatitidis*^14^. In this study, our proteomics analysis detected 70 ribosomal proteins, and 53 of them were upregulated by gamma radiation exposure, while none of them were downregulated.

Ribosomal proteins act in both individual and combined forms. Ribosomes are assembled from ribosomal RNA and ribosomal proteins, which are essential for the translation of information contained in messenger RNAs (mRNAs) into functional proteins, the ultimate step in the genetic program^17^. Although RPL3 induces G1 cell cycle arrest through the formation of protein complexes and inhibits tumor progression^18,19^, most ribosomal proteins act as tumor promoters. RPL3 also plays a significant role in the regulation of the DNA repair process^20,21^. Additionally, many ribosomal proteins act in a ribosome-free pattern: RPS13, RPS15A and RPL21 regulate the cell cycle; RPS3, RPL3, RPL6, RPL23, RPS14 and RPS26 take part in DNA repair; RPS3, RPL3, RPS15A and RPS7 control apoptosis; RPL22, RPLP0 and RPL3 are involved in endoplasmic reticulum stress and autophagy; and RPL34, RPS6, RPS3 and RPL3 affect cell migration^22^. These ribosomal proteins were upregulated by GKRS therapy in our study (Fig. 3).

Radiologically upregulated ribosomal proteins may be involved in radiation resistance and cancer progression. A large increase in ribosomal content invoked by acute gamma radiation exposure could be used to augment recovery by increasing the cell’s ability to produce proteins involved in recovery^14^. IR produces dissociated RPS3 to elevate the inflammation, proliferation, and aggressiveness of NSCLC^23,24^. RPS3 participates in the elimination of DNA damage and increases the glycosylase activity of 8-oxoguanine DNA glycosylase^20^. The interaction of RPL6 with histone H2A participates in the DNA damage–induced G2–M checkpoint, DNA damage repair, and cell survival^25^. RPS26 participates in the DNA repair process by directly regulating p53 transcriptional activity in response to DNA damage^26^. RPLP0 enhances DNA double-strand break repair and promotes radiation resistance^27^. RPS3, RPL6, RPS26 and RPLP0 accumulated after GKRS in our study (Fig. 3). Studies have shown that the relationship between the overactivation of ribosomes and tumorigenesis involves not only an increase in protein synthesis but also a change in mRNA translation patterns^28^. In general, many mRNAs encoding oncoproteins, growth factors, survival factors and cell cycle regulators have low affinity for ribosomes. Ribosomes are competitively bound by high affinity mRNA (such as housekeeping genes) when the number of ribosomes is limited^28^. Selinexor, an inhibitor of ribosomal biogenesis, decreased ribosomal biogenesis along with reductions in translational efficiency and protein synthesis in cancer cells and enhanced the radiosensitivity of cancer cells^29,30^. Therefore, inhibition of ribosomes or ribosomal biogenesis may enhance the therapeutic effect of GKRS therapy.

The validation with large-scale samples is needed. It is difficult to obtain patient tissue during the hyperacute phase of GKRS. Nearly all patients are not operated on the BMs within several months after GKRS therapy. Thanks to the implementation of clinical trial (ChiCTR2000038995), we obtained 5 samples post GKRS therapy with sufficient cancer tissues. In conclusion, BM tissue of lung cancer was obtained after the hyperacute phase of GKRS, and proteomics was used to explore the mechanism of gamma radiation. Seventy out of 134 ribosomal proteins were detected in this study. Interestingly, 53 of the 70 ribosomal proteins were upregulated by GKRS, but none of the ribosomal proteins were downregulated. We identified 7 key ribosomal proteins (RPL4, RPS19, RPS16, RPLP0, RPS2, RPS26 and RPS25) whose protein levels weakly predicted prognosis. However, the risk score determined based on these key ribosomal proteins could greatly predict the prognosis of LUAD patients. Therefore, radiotherapy combined with inhibition of ribosomal protein activation in cancer patients may be a promising treatment strategy.

## Data Availability

The datasets generated during and/or analysed during the current study are available from the corresponding author on reasonable request.
